# Inter-comparison of Seasonal Variation, Chemical Characteristics, and Source Identification of Atmospheric Fine Particles on Both Sides of the Taiwan Strait

**DOI:** 10.1038/srep22956

**Published:** 2016-03-14

**Authors:** Tsung-Chang Li, Chung-Shin Yuan, Hu-Ching Huang, Chon-Lin Lee, Shui-Ping Wu, Chuan Tong

**Affiliations:** 1 Institute of Environmental Engineering, National Sun Yat-sen University, Taiwan, Kaohsiung 804, R.O.C; 2 Department of Marine Environment and Engineering, National Sun Yat-sen University, Taiwan, Kaohsiung 804, R.O.C; 3 College of Environment and Ecology, Xiamen University, Xiamen 361005, P.R.C; 4 School of Geographic Science, Fujian Normal University, Fuzhou, P.R.C

## Abstract

The spatiotemporal distribution and chemical composition of atmospheric fine particles in areas around the Taiwan Strait were firstly investigated. Fine particles (PM_2.5_) were simultaneously collected at two sites on the west-side, one site at an offshore island, and three sites on the east-side of the Taiwan Strait in 2013–2014. Field sampling results indicated that the average PM_2.5_ concentrations at the west-side sampling sites were generally higher than those at the east-side sampling sites. In terms of chemical composition, the most abundant water-soluble ionic species of PM_2.5_ were SO_4_^2−^, NO_3_^−^, and NH_4_^+^, while natural crustal elements dominated the metallic content of PM_2.5_, and the most abundant anthropogenic metals of PM_2.5_ were Pb, Ni and Zn. Moreover, high OC/EC ratios of PM_2.5_ were commonly observed at the west-side sampling sites, which are located at the downwind of major stationary sources. Results from CMB receptor modeling showed that the major sources of PM_2.5_ were anthropogenic sources and secondary aerosols at the both sides, and natural sources dominated PM_2.5_ at the offshore site. A consistent decrease of secondary sulfate and nitrate contribution to PM_2.5_ suggested the transportation of aged particles from the west-side to the east-side of the Taiwan Strait.

The Taiwan Strait, located between Taiwan Island and mainland China, is one of the busiest marine transportation routes in Asia. Large quantities of anthropogenic air pollutants, including particulate matter, are emitted to the atmosphere and carried across the Taiwan Strait via dry/wet deposition and atmospheric dispersion. The impacts of Asian dusts, biomass burning, and Chinese haze on ambient particulate air quality commonly occur in spring and winter, when large quantities of natural and/or anthropogenic particles are driven toward and even across the Taiwan Strait.

For the past few years, there has been increasing concern about PM_2.5_ pollution around the Taiwan Strait due to its key role in atmospheric visibility degradation^1^,^2^,^3^,^4^,^5^, adverse health effects^6^,^7^,^8^,^9^,^10^,^11^, and climate change^12^,^13^,^14^,^15^,^16^. Previous studies have reported that the chemical characteristics of atmospheric aerosols correlate closely to poor ambient air quality and low atmospheric visibility^4^,^17^,^18^. The typical chemical components of fine particles, such as sulfate (SO_4_^2−^) and nitrate (NO_3_^−^), are generally the major components of secondary aerosols in the atmosphere, which are chemically converted from sulfur dioxide (SO_2_) and nitrogen oxide (NO_x_), respectively. Sulfur oxidation ratio (SOR) expresses the degree of oxidation of sulfur in terms of the ratio of non-sea salt sulfate to total sulfur (i.e., non-sea sulfate plus SO_2_). Similarly, nitrogen oxidation ratio (NOR) expresses the degree of oxidation of nitrogen in terms of the ratio of nitrate to total nitrogen (i.e., nitrate plus NO_x_). SORs and NORs higher than 0.25 and 0.1, respectively, suggest the highly potential formation of secondary inorganic aerosols in the atmosphere^19^,^20^.

To date, only a few researchers have focused on the spatiotemporal distribution, chemical characteristics, and transportation routes of atmospheric fine particulate matter across the Taiwan Strait, due to the jurisdictional differences between Taiwan and China, which result in practical difficulties for conducting strait-side researches in terms of cross-strait cooperation^21^,^22^. Previous studies have shown that particulate matter, mainly PM_2.5_, is frequently transported across the Taiwan Strait in the event of Asian duststorms during the Northeastern Monsoon periods. Accordingly, it is important to establish data on the background physicochemical characteristics of atmospheric fine particles, as well as their spatial distributions and seasonal variations given various meteorological conditions, and to further clarify the influences of atmospheric fine particles on ambient air quality for both sides of the Taiwan Strait and their transportation routes across the Taiwan Strait.

In order to ascertain the spatiotemporal variation and to characterize the chemical characteristics of atmospheric fine particles around the coastal region of the Taiwan Strait, six PM_2.5_ sampling sites including two sites in the Fujian Province, one site at the Penghu Islands, and three sites on the Taiwan Island, were selected for this particular study. The aims of this study were to investigate the spatiotemporal variation and chemical characteristics of atmospheric fine particles (PM_2.5_) in the coastal regions around the Taiwan Strait and to chart their transportation routes across the Taiwan Strait.

## Results

### Spatiotemporal Variation of PM_2.5_ Concentration

**Figure 1:**
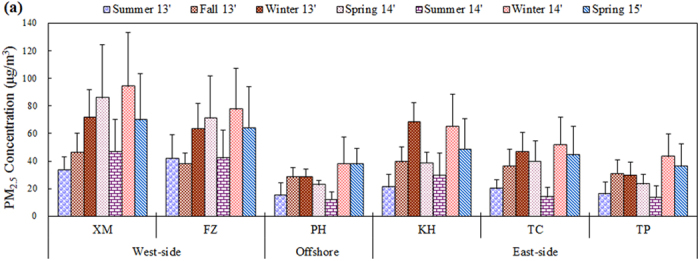
Seasonal variation of PM_2.5_ concentrations at six sampling sites around the Taiwan Strait.

The location and environmental description of six selected representative PM_2.5_ sampling sites around the Taiwan Strait are listed in Table S-1. Figure 1 illustrates the seasonal variations of PM_2.5_ concentrations, which were generally higher in winter and spring than in summer and fall. Moreover, the PM_2.5_ concentrations measured at the west-side sites were always higher than those at the east-side sites. The PM_2.5_ concentrations were significantly increased from the north sites to the south sites at the west-side and east-side sites of the Taiwan Strait. The seasonal variation of PM_2.5_ concentration was consistent at all sampling sites in this study. The lower PM_2.5_ concentration were observed at the PH and TP sites, suggested that these two sites located at the windward of Northeastern Monsoon in spring, fall, and winter. The major contribution of atmospheric fine particles (PM_2.5_) was probably from long-range transportation at the PH and TP sites. Both PH and TP sites were located at the windward areas of Taiwan Island. The Northeastern Monsoons generally blew air pollutants from northern China, Korea Peninsula, and Japan Islands. Moreover, there are very few local sources (including an oil-fired power plant, construction sites, and vehicular exhausts) at the Penghu islands. The TP site is located at the northeastern tip of the Taiwan Island as a background site under northeasterly monsoons without the interferences of local sources. The results indicated that the contribution of atmospheric fine particles (PM_2.5_) came mainly from long-range transportation rather than local emissions. On the contrary, other sampling sites were influenced by both long-range transportation and local regional emission.

Table 1 summarizes the concentrations of PM_2.5_ and PM_2.5–10_, and their ratios at the PM_2.5_ sampling sites around the Taiwan Strait. For all sites, both PM_2.5_ and PM_2.5–10_ concentrations in winter and spring were generally higher than those in summer and fall. The mass fraction of fine particles (PM_2.5_/PM_10_) ranged from 54.5% to 60.6% on the west-side, from 46.0% to 58.7% at the offshore island, and from 54.2% to 67.6% on the east-side of the Taiwan Strait, indicating that PM_10_ was dominated by the fine particle (PM_2.5_) size fraction. Higher PM_2.5_/PM_10_ ratios in winter and spring suggested the potential contribution of anthropogenic particles from both local sources and long-range transportation toward the Taiwan Strait.

The PM_2.5_ sampling results indicated that the average levels of PM_2.5_ at the west-side sites were generally higher than those at the east-side sites. According to the concentration of PM_2.5_ collected at the east-side sites, the PM_2.5_ concentration in southern Taiwan tended to be higher than that in northern Taiwan. The sampling site in Kaohsiung City (KH) is located at the remote coast nearby the biggest industrial city in southern Taiwan. There are more than 1.5 million automobiles and a total of 1,911 factories, including three utility power plants, two cement mills, an integrated steel plant, nine sizable iron works and fourteen petrochemical plants in metro Kaohsiung (153 km^2^). Moreover, Kaohsiung City is surrounded by four heavily polluted industrial parks including three petrochemical industrial parks and one iron industrial parks. As a result, the PM_2.5_ concentrations in southern Taiwan were higher than those in central and northern Taiwan. For the two east-side sites (TC and KH), the 24-h average concentration of PM_2.5_ frequently violated Taiwan’s ambient air quality standard of 35 μg m^−3^. Atmospheric PM_2.5_ levels at the FZ and XM sites were the highest among the six sampling sites, while the lowest PM_2.5_ concentration was measured at the offshore PH site, where PM_2.5_ and PM_2.5–10_ concentrations in spring and winter were generally higher than those in summer. Compared to PM_2.5–10_, PM_2.5_ accounted for the major portion of PM_10_ at all sampling sites.

### Chemical Characteristic of PM_2.5_

**Figure 2:**
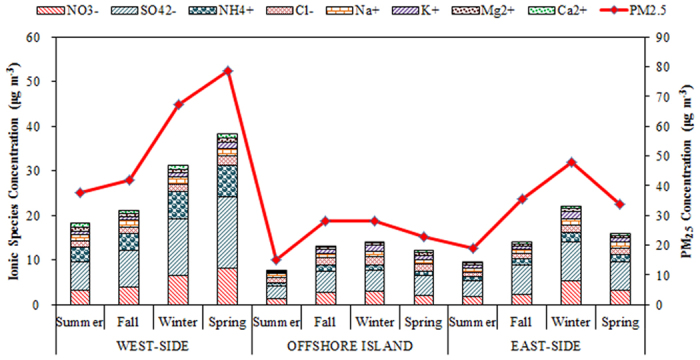
Spatiotemporal variation of ionic concentration of PM_2.5_ sampled around the Taiwan Strait.

Figure 2 illustrates the spatiotemporal variation of the water-soluble ionic species of PM_2.5_ in the areas around the Taiwan Strait. The most abundant water-soluble ionic species of PM_2.5_ were SO_4_^2−^, NO_3_^−^, and NH_4_^+^, indicating that secondary inorganic aerosols (SIA) were the major portion of PM_2.5_. The most possible inorganic compounds of PM_2.5_ were ammonium sulfate ((NH_4_)_2_SO_4_) and ammonium nitrate (NH_4_NO_3_)^23^,^24^,^25^,^26^ which were originated from the neutralization of sulfuric and nitric acids with ammonia^27^. For this particular study, the molar concentrations of Na^+^, Mg^2+^, and Ca^2+^ were relatively lower than those of NH_4_^+^, and their contribution to PM_2.5_ can be negligible. Possanzini *et al.*^28^ reported that the neutralization factor (NF) can be used to evaluate the neutralization capacity of Ca^2+^, Mg^2+^, and NH_4_^+^ with major acidic ions (SO_4_^2−^ and NO_3_^−^) (equation (1)).


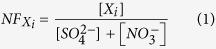


where [*X*_*i*_] represents the concentration (μeq m^−3^) of desired cations (Ca^2+^, Mg^2+^, and NH_4_^+^). The NF values of NH_4_^+^, Ca^2+^, and Mg^2+^ are listed as Table 2.

The NF values of NH_4_^+^ were significantly higher than those of Ca^2+^and Mg^2+^. The NF values of NH_4_^+^ were 2–3 times and 8–16 times higher than those of Ca^2+^and Mg^2+^ at the west-side sites, 2–4 times and 5–9 times higher than those of Ca^2+^and Mg^2+^ at offshore site, and 3–5 times and 5–8 times higher than those of Ca^2+^and Mg^2+^ at east-side sites, respectively. It suggested that the acidic ions in PM_2.5_ were mainly neutralized by NH_4_^+^. Moreover, the secondary inorganic aerosols (SO_4_^2−^, NO_3_^−^, and NH_4_^+^) accounted for 56.6–83.0% of water-soluble ions and 23.0–39.0% of PM_2.5_, respectively. These results further indicated that NH_4_^+^ was the major portion of secondary inorganic aerosols (SIA) in PM_2.5_, which concurred with previous studies^29^,^30^,^31^.

The SO_4_^2−^ concentrations, particularly at the FZ and XM sites, were higher than those at the TP, TC, KH, and PH sites. The concentrations of water-soluble ionic species of PM_2.5_ at the west-side sites were generally higher than those at the east-side and the offshore sites, suggesting that the PM_2.5_ concentration was highly influenced by local anthropogenic sources on the west-side of the Taiwan Strait, due to the rapid economic development of the coastal region of Fujiang Province in Southeast China. The variation of water-soluble ionic concentration was with that of PM_2.5_ concentration. Previous studies showed that the higher PM_2.5_ concentrations in spring were mainly attributed to the combination of the relatively elevated emissions from fossil fuel (e.g. coal) combustion and biomass burning (e.g. woods) for space heating, long-range transportation of air pollutants from North and East China in spring^32^,^33^.

Table 3 summarizes the mass ratios of secondary inorganic aerosols to water-soluble ions and PM_2.5_ concentration (i.e. SIA/WSI and SIA/PM_2.5_) during the sampling periods. The secondary inorganic aerosols accounted for 56.6–83.0% of WSI and 23.0–39.0% of PM_2.5_, respectively. Similarly, the concentrations of SIA on the west-side were mostly higher than those on the east-side of the Taiwan Strait. At the west-side sites, the concentrations of SO_4_^2−^ and NO_3_^−^ were significant higher in spring, and approximately 1.5–2.0 times higher than other sites. We speculated that the secondary SO_4_^2−^ and NO_3_^−^ were mainly attributed to the burgeoning industrial development in the coastal region of southeastern China. Huang *et al.*^34^ indicated that the sources of SIA are relatively well constrained: in urban areas, sulfate forms primarily through atmospheric oxidation of SO_2_ emitted mainly from coal burning, while nitrate derives from NOx emitted mainly from vehicle exhaust and power plants.

On the contrary, the highest amounts of water-soluble SO_4_^2−^ and NO_3_^−^ were observed in winter at both the offshore site and the east-side sites. This suggested that anthropogenic emissions from northern China could migrate toward the Taiwan Strait during the Northeastern Monsoon periods. The contributions of oceanic spray were generally higher in islands and coastal regions. Previous study (Zhuang *et al.*^35^) reported that the amount of nitrate associated with soil particles ([NO_3_^−^]_soil_) can be estimated by subtracting the amount of nitrate associated with sea-salt particles ([NO_3_^−^]_sea-salt_) from the total nitrate ([NO_3_^−^]). Assuming that chloride depletion is caused by both nitrate and excess sulfate, the amount of nitrate associated with sea-salt equals the amount of chloride depleted minus the amount of sulfate formed on sea-salt particles. The amount of nss-[NO_3_^−^] can be determined by the following equation (2)^35^.


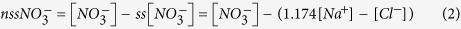


Table 4 summarizes the nss-NO_3_^−^, ss-NO_3_^−^, and ratio of nss-NO_3_^−^/NO_3_^−^ at the Taiwan Strait. This study revealed that the nss-NO_3_^−^ concentrations ranged from 3.0 to 6.1 μg m^−3^ at the west-side sites, from 0.9 to 5.8 μg m^−3^ at the offshore site, and from 1.2 to 4.2 μg m^−3^ at the east-side sites, respectively. The ratios of nss-NO_3_^−^/NO_3_^−^ ranged from 71.4% to 78.4% at the west-side sites, from 70.4% to 82.1% at the offshore site, and from 63.3% to 73.8% at the east-side sites, respectively.

NO_3_^−^, SO_4_^2−^ and NH_4_^+^ are associated together in the same particulate system in the likely form of NH_4_NO_3_, and [NH_4_]_2_SO_4_ or NH_4_HSO_4_. Particulate phase NH_4_^+^ concentrations can be calculated using the stoichiometric ratios of different compounds and compared with actual measurements^28^. Nitrate is in the form of NH_4_NO_3_, while sulfate is in the forms of either (NH_4_)_2_SO_4_ or NH_4_HSO_4_ which can be estimated by equations (3) and (4)^36^.


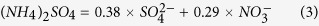



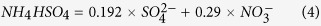


**Figure 3:**
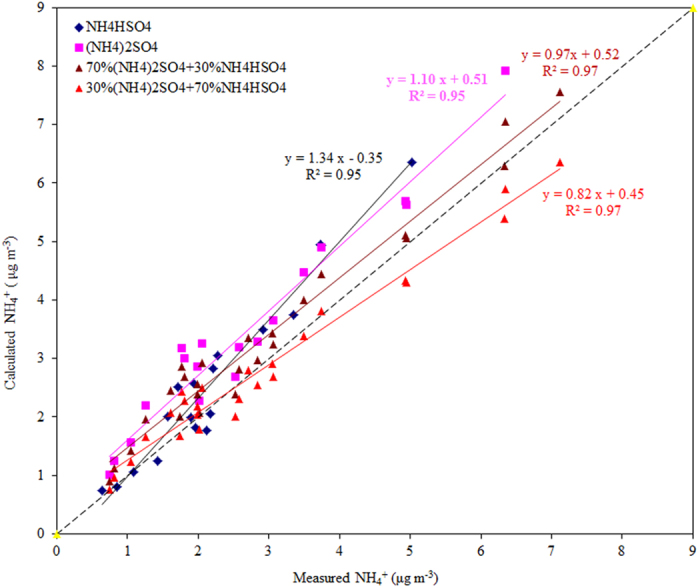
Comparison between calculated and measured ammonium (NH_4_^+^) in the PM_2.5_ particulate system.

As shown in Fig. 3, the calculated ammonium from (NH_4_)_2_SO_4_ or NH_4_HSO_4_ was higher than the measured ones, suggesting that fine particles collected during the sampling period were either more acidic, or that sulfate and nitrate were associated with cations other than NH_4_^+^, such as Na^+^ and K^+^. Previous study used the above equations to calculate the relationship of NO_3_^−^, SO_4_^2−^, and NH_4_^+^. Ammonia is known to neutralize sulfuric acid irreversibly, and then nitric acid. Additionally, hydrochloric acid may react with gaseous ammonia to form ammonium chloride aerosols. However, in thermodynamic equilibrium conditions ammonium chloride is reported to be 2–3 times more volatile than ammonium nitrate^37^ and its formation occurs later. Thus, ammonia was believed to be neutralized firstly by sulfuric acid to form ammonium sulfate and/or ammonium bisulfate^38^,^39^,^40^. In this study, we assumed that both sulfate (SO_4_^2−^) and bisulfate (HSO_4_^−^) could be neutralized by ammonia with various portions. However, the calculated ammonium concentrations were significantly above the 1:1 line for PM_2.5_ (see Fig. 3). We intended to find an accurate relationship between the calculated and measured ammonium that approaches 1:1 line. Finally, the correlation among inorganic sulfate and bisulfate appears to be the combination of 70% (NH_4_)_2_SO_4_ and 30% NH_4_HSO_4_. Moreover, we also try other portion ratios (e.g. 60% (NH_4_)_2_SO_4_ + 40% NH_4_HSO_4_ and 40% (NH_4_)_2_SO_4_ + 60% NH_4_HSO_4_) to fit the 1:1 line. However, the relationship with 60% (NH_4_)_2_SO_4_ + 40% NH_4_HSO_4_ and 40% (NH_4_)_2_SO_4_ + 60% NH_4_HSO_4_ were both inferior to 70% (NH_4_)_2_SO_4_ + 30% NH_4_HSO_4_ for this particular case. Therefore, we selected the portion of 70% (NH_4_)_2_SO_4_ + 30% NH_4_HSO_4_ in order to fit the 1:1 line for PM_2.5_. The correlation among these two major inorganic sulfates appears to be the combination of 70% (NH_4_)_2_SO_4_ and 30% NH_4_HSO_4_.

**Figure 4:**
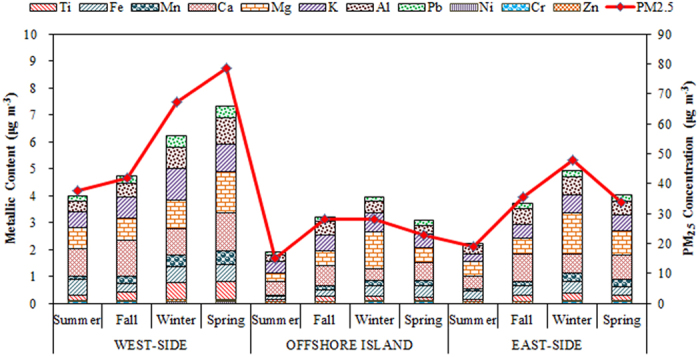
Spatiotemporal variation of metallic concentration of PM_2.5_ sampled around the Taiwan Strait.

Figure 4 illustrates the spatiotemporal variation of metallic elements of PM_2.5_ in the areas around the Taiwan Strait. The most abundant metallic elements of PM_2.5_ were crustal elements (Al, Fe, Na, Mg, K, and Ca) and followed by anthropogenic elements (Zn, Ni, and Pb). The highest metallic concentrations of PM_2.5_ were commonly observed at the west-side sites, as compared to those at the east-side and offshore sites. Among the crustal elements, Ca, Al and Fe were the most abundant metals, with a much higher concentration of Ca than the other crustal elements. Al and Ca are the main elements in the Earth’s crust, and they come from the wind-blown soil dusts and fugitive dusts generated at the construction sites; both of which increase the loading of atmospheric particles^41^. The concentrations of anthropogenic elements (Zn, Ni, and Pb) at the west-side sites were also higher than those at the east-side sites. Zn and Pb were the major components among the anthropogenic elements. Previous studies reported that the high traffics of vessels navigating in the Taiwan Strait resulted in high Zn and Pb emissions^21^,^22^. The oil burning on heavy vessels is one of the major sources of Pb, which makes it a good marker for vessel exhausts. In addition to vessel exhaust, Pb is emitted mainly from incinerators^42^,^43^.

**Figure 5:**
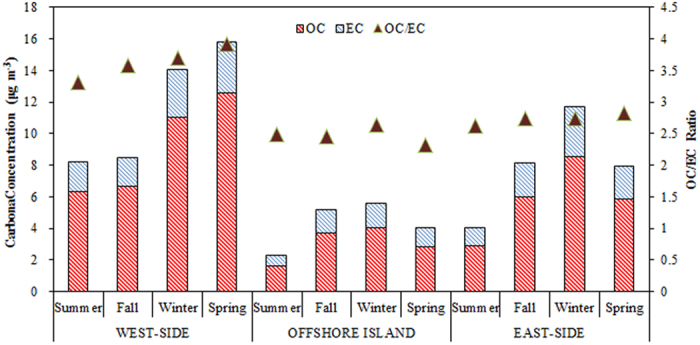
Spatiotemporal variation of carbonaceous concentration and their mass ratios (OC/EC) of PM_2.5_ sampled surrounding the Taiwan Strait.

Figure 5 illustrates the spatiotemporal variation of carbonaceous contents and their mass ratios (OC/EC) of PM_2.5_ sampled around the Taiwan Strait. Carbonaceous compounds in the atmosphere mainly consist of elemental and organic carbons (EC and OC). EC is a product of incomplete combustion from residential coal, motor vehicle fuel, and biomass burning. OC originates from primary anthropogenic sources like above mentioned combustions and from the formation of secondary OC by chemical reactions in the atmosphere. The concentrations of OC in PM_2.5_ were always higher than those of EC for all seasons in the areas around the Taiwan Strait. Carbon concentrations were higher in some of coastal sites, especially at the XM and FZ sites, and followed by the east-side sites and the offshore site. Among the six sampling sites, XM had the highest average OC (10.2 ± 2.4 μg m^−3^) and EC (3.4  ±  1.3 μg m^−3^) concentrations in spring. Xiamen is a heavily industrial city and traffic emissions surrounding the Xiamen Bay. The results indicated that the concentration levels of carbonaceous particles in Xiamen were higher due to a large amount of carbon emissions from anthropogenic sources and human activities. The lowest OC averages were found at the PH site (1.6 ± 0.5 μg m^−3^) in summer. The PH site located at an offshore island where clean marine air can dilute PM_2.5_ from long-range transportation, resulting in local emission accumulation and lower OC and EC levels at the Penghu Islands. The concentrations of OC and EC were always lower in summer and higher in winter at all the sampling sites. Wintertime OC and EC concentrations were about two to three times than those in summer. The average TC concentrations among six sites were in order of XM (11.8 μg m^−3^) > FZ (10.8 μg m^−3^) > KH (9.3 μg m^−3^) > TC (6.1 μg m^−3^) > TP (3.5 μg m^−3^) > PH (4.2 μg m^−3^). Because the concentrations of TC were mainly dependent upon both emission amounts and meteorological conditions in spring and summer, the TC concentration increased from the south to the north and its variation was consistent quite well with the PM_2.5_ concentration.

The OC/EC ratios at the sampling sites ranged from 2.9 to 3.8 on the west-side sites, from 1.7 to 3.3 on the east-side sites, and from 1.7 to 3.6 at the offshore site. The highest OC/EC ratios of PM_2.5_ were observed at the XM site, which was located at the downwind of major stationary sources, such as petrochemical and mechanical industries, light industries, ceramics and stone process industries, and textile industries. High OC/EC ratios of PM_2.5_ were observed at the west-side sites, which were adjacent to major at the west-side sites, which were adjacent to major emission sources, such as industrial boilers, heavy oil, and coal burning. Under certain meteorological conditions (e.g., Northeastern Monsoons), emissions of huge amounts of volatile organic compounds (VOCs) from various sources (e.g., the textile plants at the Jinjing River Basin) contribute to the formation of secondary organic aerosols (SOA). The OC/EC ratio has been used in previous studies to determine whether the transformation and emission properties of carbonaceous aerosol^44^. A high OC/EC ratio coupled with a poor correlation implies an influx of urban pollutants from elsewhere or the formation of secondary OC (SOC) from photochemical reactions. On the other hand, a high correlation indicates primary emission and a secondary formation derived from the primary carbon^45^,^46^.

**Figure 6:**
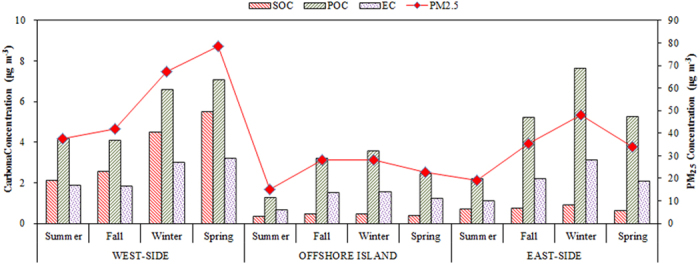
Seasonal variations of estimated POC, SOC, and EC concentrations at all sampling sites.

Figure 6 illustrates the spatiotemporal variation of the estimated secondary OC (SOC), primary OC (POC), and elemental carbon (EC) concentrations of PM_2.5_ sampled around the Taiwan Strait. The concentrations of secondary organic carbon (SOC) were estimated using the EC-tracer method^46^. Moreover, the primary OC to EC ratio, (OC/EC)_pri_, was simplified as the minimum ratio of particulate OC to EC in each season, representing primary OC and EC emissions. If the primary OC/EC ratio ((OC/EC)_pri_) is available, we can then determine the SOC and POC concentration of PM_2.5_ by equations (5) and (6),


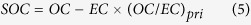



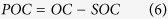


However, the (OC/EC)_pri_ is a source- and seasonal-specific parameter, and is also affected by the carbon determination method^47^. The observed minimum OC/EC ratio at a specific sampling site was often used to represent the (OC/EC)_pri_ assuming that the meteorological conditions are not favorable for the SOC formation during the sampling periods^47^,^48^. This study estimated the primary OC/EC ratio ((OC/EC)_pri_) as the minimum ratio of particulate OC to EC in each season at all sampling sites, representing the primary OC and EC of PM_2.5_^49^,^50^.

The mass concentrations of POC, SOC, and OC in PM_2.5_ at all sampling sites are shown in Fig. 6. The average POC concentrations in west-side sites ranged from 2 to 3 times higher than those at the east-side sites. Previous study reported that primary OC are emitted directly from combustion, while secondary OC is mainly produced by gas-to-particle conversion or chemical reaction^51^. In this study, the primary OC to EC ratio, (OC/EC)_primary_, was simplified as the minimum ratio of particulate OC to EC (OC/EC)_min_ at each sampling sites of the Taiwan Strait in each season to estimate the SOC concentrations. In fact, at the high pollution metropolitan sites (i.e. XM, FZ, and KH sites), the (OC/EC)_min_ were generally higher than those at the background sites (i.e. PH and TP sites), due to high anthropogenic emissions in these regions. The results indicated that overestimation of (OC/EC)_min_ could result in higher (OC/EC)_min_ and the lower SOC concentrations at the XM, FZ, and KH sites. The higher POC concentrations were generally overestimated at the west-side sites than those at the east-side sites, except for the KH sites. The highest average SOC concentrations and ratio of SOC/OC occurred in fall and summer at the west-side sites. A similar trend of the highest average concentrations of SOC was observed in winter (0.7 μg m^−3^), while the highest ratios of SOC/OC were 23.4% and 34.4% at the offshore site and the east-side sites, respectively. The lower SOC/OC in winter and spring resulted mainly from the higher (OC/EC)_pri_ (2.2) than those in summer (1.5) and fall (1.2) at all the sampling sites in this study. Previous study indicated that SOC is mainly formed from the oxidation of volatile or semi-volatile organic compounds and is prevalent in high photochemical oxidation seasons (i.e. summer), which was consistent with findings of recent study in China^52^.

### Source Indicators of PM_2.5_ Chemical Composition

There are several ratios of chemical species can be used as valuable indicators to appoint the particles from specific sources^2^,^53^,^54^. Previous researches reported that the mass ratios of EC/TC, K^+/^TC, and TC/SO_4_^2−^ can be used to identify the sources mainly from biomass burning^41^. When the mass ratio of EC/TC ranges from 0.1 to 0.2, K^+^/TC ranges from 0.5 to 1.0, and TC/SO_4_^2−^ ranges from 6 to 15, it suggests that the sources were mainly contributed from biomass burning. The mass ratios of NO_3_^−^/nss-SO_4_^2−^ have also been used to evaluate the contributions from stationary and mobile sources^2^,^55^. The mass ratios of NO_3_^−^/nss-SO_4_^2−^ higher than unity indicated that the sources of particles were mainly from mobile sources. Conversely, the mass ratios of NO_3_^−^/nss-SO_4_^2−^ lower than unity suggested that the sources of particles mainly came from stationary sources.

Table 5 compares the mass ratios of major chemical species during the sampling periods. In this study, the mass ratios of EC/TC, K^+^/TC, NO_3_^−^/nss-SO_4_^2−^, and TC/SO_4_^2−^ were 0.3, 0.2, 0.6, and 0.9 at the XM site, respectively, while similar trend were observed at the FZ site (0.3, 0.1, 0.7, and 1.2, respectively). The results indicated that high SO_4_^2−^ and NO_3_^−^ concentrations observed at the west-side sites and KH site were mainly from stationary sources due to burgeoning industrial development in the coastal region. At the east-side sites, the biomass burning indexes (EC/TC, K^+/^TC, and TC/SO_4_^2−^) and stationary/mobile source (NO_3_^−^/nss-SO_4_^2−^) were in the range of ranged 0.4–0.4, 0.1–0.2, 0.8–0.9, and 0.5–0.8, respectively. According to the reports from VanCuren^55^, high SO_4_^2−^ and NO_3_^−^ concentrations came mainly from stationary sources.

### Spatiotemporal Distribution of PM_2.5_ Chemical Composition

**Figure 7:**
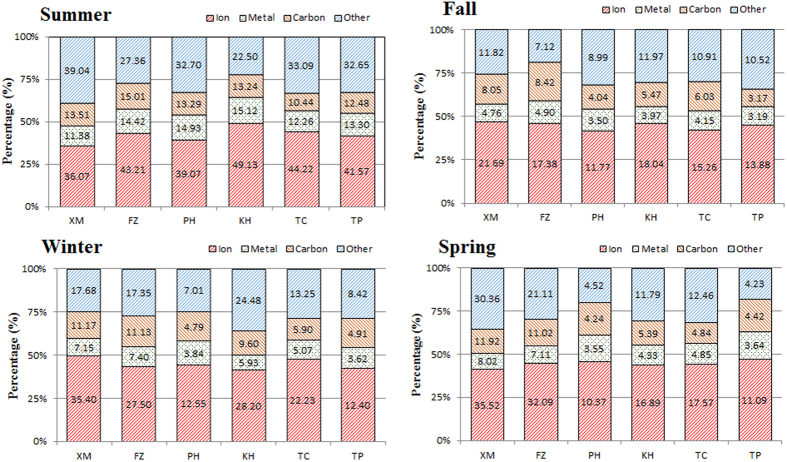
Spatiotemporal variation of mass percentage of PM_2.5_ chemical compositions surrounding the Taiwan Strait.

Figure 7 illustrates the spatiotemporal distribution of the mass percentage of chemical composition of PM_2.5_ at the sampling sites around the Taiwan Strait. In summer, the chemical fingerprints (especially metallic elements) were consistent at the FZ and XM sites on the west side of the Taiwan Strait, but not at the KH site. The percentages of ionic species and metallic elements at the KH site were the highest for the sampling sites. Same trend in chemical composition could be influenced by local emissions from nearby XM, FZ, and KH sites in urban areas. The distributions of the chemical composition were most similar at the TP, TC, and PH sites, suggesting that the sites located at the windward areas of Taiwan had similar chemical compositions of PM_2.5_. In fall, the percentages of carbonaceous content were higher than those in summer, especially at the sites on the west side of the Taiwan Strait, due to their proximity to major emission sources along the coastal regions of the southeastern China. During the Northeastern Monsoon periods, emissions of huge amounts of particulates from various sources (e.g., textile plants at the Jinjing River Basin) could result in the higher percentages of ionic and carbonaceous contents. The distributions of chemical composition were most similar at the TP and PH sites. In winter, the concentrations of chemical species were higher than those in summer and fall, especially for carbonaceous content. The TP and PH sites had quite similar chemical fingerprints. The highest percentages of ionic species were also observed at the XM, FZ, and TC sites. Higher percentages of ionic species at the XM and FZ sites suggested that the XM site was adjacent to major stationary sources, such as petrochemical plants and industrial complexes in the southeastern coastal region of China. The percentages of ionic species at the west-side sites, the offshore site, and the east-side sites were similar in spring, but the highest percentage of carbonaceous content was observed at the TP and TC sites, suggesting that the Northeastern Monsoons could bring secondary carbonaceous particles from the coastal region of southeastern China.

### Reconstruction of PM_2.5_

PM_2.5_ can be calculated by material balance equation for gravimetric mass^56^. In this study, the chemical composition of PM_2.5_ could be divided into nine major parts: nitrate (NO_3_^−^), sulfate (SO_4_^2−^), ammonium (NH_4_^+^), chloride (Cl^−^), organic matter (OM), elemental carbon (EC), soil dusts, trace elements and others. Organic material (OM) was estimated from an organic carbon (OC) multiplier (f) that accounts for unmeasured hydrogen (H), oxygen (O), nitrogen (N), and sulfur (S) in organic compounds^53^. Multipliers of 1.4 to 1.8 have been found to best represent the complex mixture of organic molecules in OM. A factor of 1.6 in converting OC to OM was used in this study according to previous results^57^,^58^,^59^,^60^,^61^. The crustal species is estimated using a method provided by Han *et al.*^62^ (Crustal materials = CM = 1.89*Al + 2.14*Si + 1.4*Ca + 1.43*Fe + 1.66*Mg + 1.67Ti). Sea salts was estimated using the method provided by Chow *et al.*^56^ (Sea salts = [Na^+^] + [Cl^−^]). Therefore, PM_2.5_ concentrations were reconstructed by using the following equation (7),


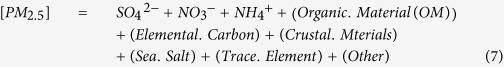


**Figure 8:**
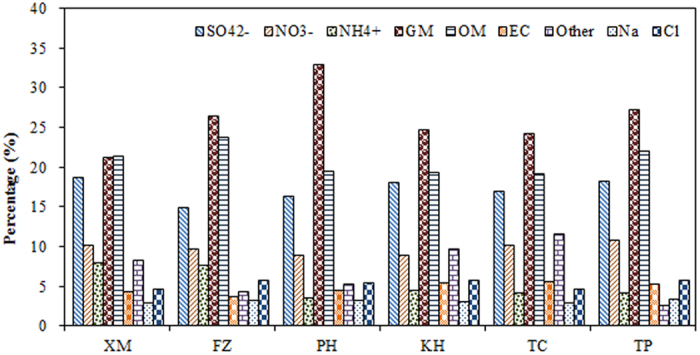
Major chemical components of PM_2.5_ at six sampling sites.

Figure 8 illustrates the material balance of PM_2.5_ at six sampling sites. Consistent with previous studies, organic materials (OM), crustal materials (CM), SO_4_^2−^, and NO_3_^−^ were the dominant components for the reconstructed PM_2.5_. The contribution of crustal materials was ordered as PH > TP > FZ > KH > TC > XM. The results indicated that CM was higher at the offshore islands in the Taiwan Strait. The contributions of OM at the west-side sites were generally higher than those at the east-side sites. Similar seasonal variation trends have also been observed for the distributions of SO_4_^2−^ and NO_3_^−^ in the Taiwan Strait. The crustal materials were higher at the PH site on the offshore islands and at the TP site in northern Taiwan. The contribution to PM_2.5_ in the Taiwan Strait was ordered as CM > OM > SO_4_^2−^ > NO_3_^−^ > EC > Cl > Na.

Table 6 compares the distribution of major chemical components of PM_2.5_ at the coastal sites around the Taiwan Strait and East China Sea. The results indicated that SO_4_^2−^, NO_3_^−^, crustal materials, and organic materials are important components in PM_2.5_ at all coastal sites. The contribution of sulfate, nitrate, and organic materials were similar to most of the other coastal sites. The contributions of organic materials to PM_2.5_ in this study (20.8~28.8%) were slightly lower than those of previous references (21.8–34.0%). It is probably attributed to the fact that the concentrations of carbonaceous content measured by thermal optical reflectance (TOR)^2^ and thermal optical transmittance (TOT)^32^ were generally higher than those measured by elemental analyzer (EA)^21^,^22^.

### Chemical Transformation of SO_2_ and NO_X_

**Figure 9:**
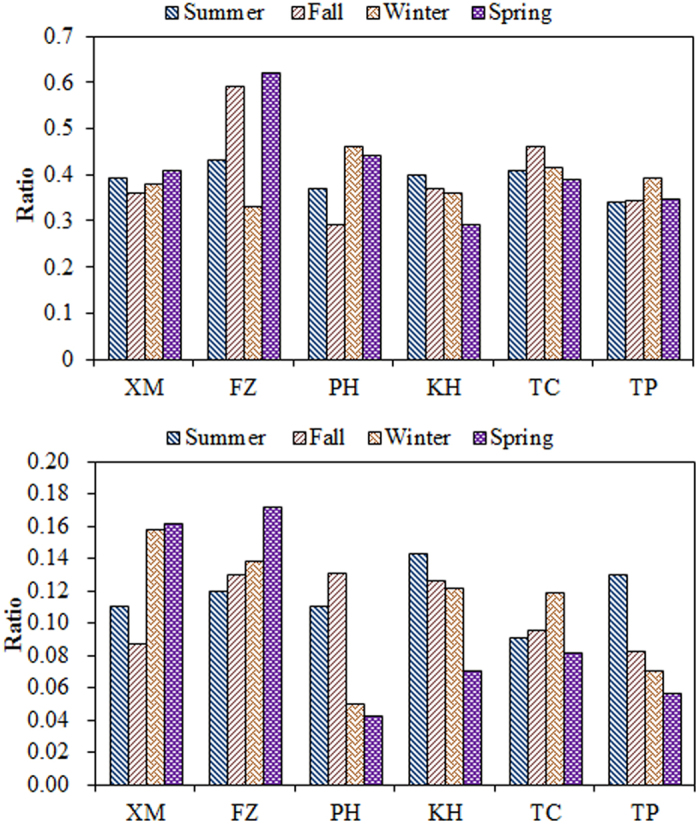
The SOR and NOR ratios of PM_2.5_ sampled around the Taiwan Strait.

The chemical transformations of SO_2_ to SO_4_^2−^ (SOR) and NO_X_ to NO_3_^−^ (NOR) for PM_2.5_ are shown in Fig. 9 (Definition please see Supplementary Information). Previous studies have reported that SOR and NOR values are less than 0.25 and 0.10, respectively, for primary pollutants, while the chemical oxidation of SO_2_ and NO_X_ forming SO_4_^2−^ and NO_3_^−^ could occur in the atmosphere when SOR and NOR values are greater than 0.25 and 0.10, respectively^20^. The average SOR values at each sampling site were generally higher than 0.25 at all seasons, especially at the XM and FZ sites. It has been reported that SOR values smaller than 0.1 represented primary source emissions, whereas SOR values above 0.25 occur when sulfate is mainly produced through the secondary transformation of SO_2_ oxidation^20^. The SOR reached up to 0.6 in spring at the FZ site mainly due to secondary pollution, suggesting that the secondary transformation came from northeastern China. In this study, high SOR and NOR values directly resulted in large amounts of secondary sulfate and nitrate particles formed in the atmosphere at the FZ site. However, micrometeorological conditions could also play an important role in the formation processes of secondary aerosol particles. The average NOR values were in the range of 0.1 to 0.2. The highest NOR was observed at the west-side sites mostly in winter and spring. The high SOR and NOR in the areas around the Taiwan Strait obtained in this study suggested that the formation of SO_4_^2−^ and NO_3_^−^ from SO_2_ and NO_X_ commonly occurred in the atmosphere.

High SOR and NOR were frequently observed on the west-side of the Taiwan Strait in winter and spring. These two ratios exhibited a similar seasonal trend at the west-side sites of the Taiwan Strait, which was generally low in summer and high in fall, winter, and spring. The SOR ratios were always higher than 0.25 at all sites around the Taiwan Strait. Noticeably, lower NOR ratios were observed at the offshore and the east-side sites of the Taiwan Strait mostly in spring and winter, due to unfavorable conditions such as low concentrations of NO_x_ for the homogeneous and heterogeneous gas-particle conversion of NO_x_^63^.

### Enrichment Factors for Metallic Elements of PM_2.5_

**Figure 10:**
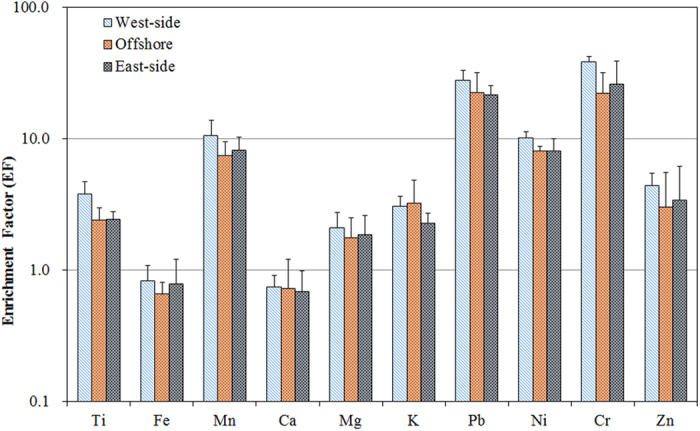
Enrichment factors of ten metallic elements to Al as the reference element across the Taiwan Strait.

To identify whether the presence of certain metallic elements in aerosols were primarily due to natural or anthropogenic processes, the enrichment factor (EF) of each metallic element was determined. Enrichment factors (EFs) are generally used to determine the degree of enrichment of a given element compared to the relative abundance of that element in the crustal components. EFs were used as the first step in evaluating the influences of crustal sources on the components of PM_2.5_^64^,^65^. In this study, aluminum (Al) was used as the reference element for crustal particles for the EF calculations^65^. The non-crustal EF was estimated using the following expression (8):


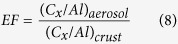


where (C_x_/Al)_aerosol_ represents the concentration ratio of specific metallic element (C_x_) to Al in aerosol; and (C_x_/Al)_crust_ represents the corresponding ratio of specific metallic element (C_x_) to Al in crustal matter. The enrichment factor has been applied to identify the source region from Inner Mongolia to the Taiwan Strait^66^. If EF ≦ 10, it is considered that the elements in the aerosols have a significant crustal and/or marine contribution and, hence, are termed non-enriched elements; whereas, if EF is greater than 10, the elements are considered to have an important proportion of non-crustal sources and, hence, are termed as enriched elements^63^. In this study, the EF values of 10 metals were in the range of 0.1 to 10, and so highly relevant, as illustrated in Fig. 10. Pb and Cr were moderately enriched (10 < EF < 100). Previous studies have reported that an EF > 10 indicates that the metallic elements have an important proportion of non-crustal sources and that a variety of emission sources could contribute to their loading in the ambient air.

The mean EFs of Ti, Mn, Mg, K, Ni, and Zn in PM_2.5_ sampled at six sampling sites ranged from 1 to 10, and the EF values for different samples were relatively constant, suggesting that these metallic elements would be more likely originated from natural sources and had no obvious enrichment in PM_2.5_. In comparison, the average EFs of Pb and Cr were in the range of 10–100, suggesting that these two elements were mainly originated from anthropogenic sources^67^. Therefore, Cr and Ni in coarse particles were thought to have more soil-related origins, while Cr and Ni in fine particles were mostly from anthropogenic combustion sources. Notably, Pb mainly came from exhaust emissions (motor/ship) might not be a major source of Pb in PM_2.5_ in the Taiwan Strait because leaded gasoline had been phased out since 2000 in Taiwan. Particulate Pb emissions were mainly came from motor vehicles^68^,^69^,^70^,^71^ for using leaded gasoline. However, after leaded gasoline being phased out since 2000, the particulate Pb could probably come from incinerators, coal-fired boilers^72^, metal brake wear^73^, and industrial activities such as metallurgical processes^74^. Moreover, the Pb might be also fugitively emitted from paved and unpaved roads where roadside leaded dusts are originally deposited by leaded particles exhausted from tail gases of motor vehicles^72^.

### Transportation Routes of Air Masses

The mass concentrations of PM_2.5_ measured at the sampling sites varied with season and wind direction. Higher PM_2.5_ concentrations were mostly observed in winter (northeasterly winds) than in summer (southwesterly winds). In order to trace the routes of air masses, backward trajectories from a specific receptor site are commonly used to identify their source regions^75^,^76^,^77^. The Hybrid Single-Particle Lagrangian Integrated Trajectory (HYSPLIT) is a widely used model that plots the trajectory of a single air parcel from a specific location and height above ground over a period of time. The 120-hour backward trajectories started at the XM, PH, and KH sites at the altitude of 100, 350, and 500 m above sea level, respectively. The level of PM_2.5_ concentrations in spring and winter was much higher than those in fall and summer, indicating that atmospheric PM_2.5_ was affected by meteorological condition. The transportation routes of air masses toward the Taiwan Strait are summarized in Table S-2 and Fig. S-1. Table S-3 summarized the concentrations of PM_2.5_ for various transportation routes.

### Source Apportionment of PM_2.5_

**Figure 11:**
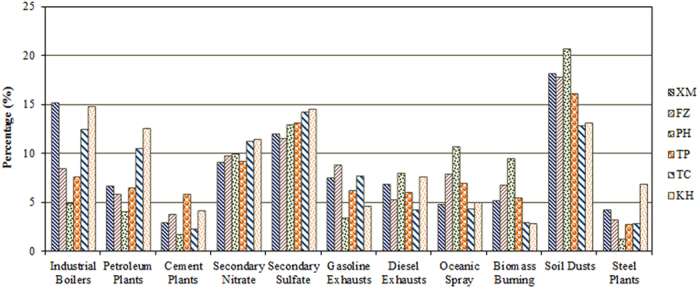
The source apportionment of atmospheric PM_2.5_ during the sampling periods.

The principle of chemical mass balance (CMB) and source Profiles of PM_2.5_ used for chemical mass balance receptor modeling are summarized at Table S-3. Figure 11 summarizes the source apportionment of PM_2.5_ collected at all sampling sites in this study, and an obvious seasonal variation was observed. According to the results of source apportionment, soil dusts (including road dusts) and industrial boilers were the main source of PM_2.5_ at the XM and KH sites, and followed by vehicular exhausts, secondary aerosols, petrochemical plants, oceanic spray, and biomass burning.

Among these sources, soil dusts, vehicular exhausts, secondary aerosols, and industrial boilers were the major sources of PM_2.5_ at the XM site, whereas soil dusts and secondary aerosols dominated PM_2.5_ at the FZ site. The contribution of industrial boilers to PM_2.5_ at the XM and KH sites was always higher than that at other sampling sites, since metro Xiamen and Kaohsiung has the largest industrial complexes compared to other sites. Although industrial boilers were the dominant sources, vehicular exhausts, and secondary aerosols were also the significant sources causing an increase in PM_2.5_ around the Taiwan Strait. Previous study reported that the Northern Monsoons transported suspended particles from the upwind emission sources (e.g., textile plants in the Jinjiang River Basin) to the downwind sites at the Kinmen Islands, causing a significant increase in secondary inorganic aerosols mainly composed of sulfate and nitrate. The source apportionment of PM_2.5_ at the FZ site located at the northwestern Taiwan Strait indicated that soil dusts, vehicular exhausts, and secondary aerosols were the main sources of PM_2.5_. Oceanic spray at the PH site contributed 6–8% higher than those at the coastal sites. Soil dusts at the Penghu islands contributed about 20% more than those at the coastal sites. High percentages of anthropogenic sources were commonly observed at the west-side sites than those at the east-side sites and the offshore site. High percentage of natural sources were observed at offshore site than those at the west-side and east-side sites, while secondary sources at the east-side sites were higher than those at the offshore site and the west-side sites. The results of the CMB models indicated that the source apportionment was divided into four different types of sources in the investigated areas, which includes anthropogenic sources, natural sources, secondary sources, and mobile sources.

Comparison of source apportionment at both sides of the Taiwan Strait indicated that higher percentage of anthropogenic sources (such as industrial boilers, petroleum plants, and steel plants) was always observed at the west-side and the east-side sites. The lowest contributions of industrial sources were always observed at the offshore islands. The results indicated that stationary emissions were the most important sources at both sides of the Taiwan Strait, suggesting the potential contribution of local sources and long-range transportation at the west-side and east-side areas of the Taiwan Strait. The higher percentage of natural sources were generally observed at the offshore islands sites, which had less industrial sources and lower human activities at the Penghu islands. The results indicated that crustal emissions were one of major sources, suggesting that the contribution of anthropogenic sources at the offshore islands were significantly lower than those at the coastal areas. Secondary inorganic aerosols (i.e. secondary sulfate and nitrate) followed the trend of east-side sites > offshore site > west-side sites, suggesting an obvious transportation phenomenon of aged particles from the west-side to the east-side of the Taiwan Strait. Higher concentrations of PM_2.5_ blown from the southeastern China experienced rapid industrial development were commonly observed, which blew air masses from the west-side to the east-side of the Taiwan Strait in fall and winter (See Table S-3). The main trajectories toward the west-side of the Taiwan Strait came from northern directions, which transported industrial emissions and secondary aerosols from the southeastern China and the coastal of the East China. Conversely, the air masses toward the east-side of the Taiwan Strait came mainly from northern China, Korea Peninsula, Japan Islands, which took longer range and duration across the East China Sea toward the east-side and the offshore islands of the Taiwan Strait. As a result, aged secondary particles could be formed during the transportation process at the east-side and the offshore islands rather than those at the west-side sites. The results indicated that the sources of PM_2.5_ were not only from northern China, but also from southern China. Different trajectories could the transport aged particles from different upwind regions toward the west-side and the east-side of the Taiwan Strait, respectively. Moreover, the contributions of vehicular exhausts at the west-side were higher than those at the offshore and the east-side sites. Vessels are mainly powered by diesel engine. Previous studies estimated the vessel emissions from fuel-based consumption or activity-based methods that commonly adopted for vessel emission inventory^78^. However, the source profile of vessel sources is lack in the Taiwan Strait. Thus, we focused mainly on vehicular exhausts covering both gasoline and diesel exhausts. We have summarized the contributions of gasoline and diesel exhausts in Table 7. The emission of diesel exhausts were generally included heavy-duty trucks and vessels for both sides of the Taiwan Strait. The contribution of diesel exhausts generally accounted for 4–8% at both side sites of the Taiwan Strait. Higher contributions of diesel exhausts were always observed at the PH, KH, and XM sites where KH and XM sites are located at the commercial harbors. Both container and merchant ships emitted lots of PM_2.5_ from diesel engines inside and outside harbors. The PH site was adjacent to a fishery port along the coastline of the Penghu Islands. Therefore, the highest contribution of diesel exhausts was always observed at the PH site. Nevertheless, the vessel exhausts were always lower than the vehicular exhausts.

## Discussion

This study investigated the spatiotemporal distribution of PM_2.5_ at the coastal and offshore islands around the Taiwan Strait. The spatial distribution of PM_2.5_ concentrations showed a consistent decrease from the west to the east and increased from the north to the south of the Taiwan Strait. The annual averaged concentration of PM_2.5_ ranged from 37.7 to 78.6 μg m^−3^ at the west-side sites, 15.1 to 28.3 μg m^−3^ at the offshore site, and 19.01 to 48.00 μg m^−3^ at the east-side sites around the Taiwan Strait, respectively, which exceeded the Air Quality Standards of Taiwan (35 μg m^−3^) and China (35 μg m^−3^ annual arithmetic average and 75 μg m^−3^ for 24-hr standard), and World Health Organization air quality guideline (10 μg m^−3^). Additionally, the PM_2.5_ concentrations in the coastal region were always higher than those at the offshore islands. The results indicated that local, regional, and long-range transportation influencing the particulate air quality of the Taiwan Strait were quite different at the west-side, offshore islands and east-side sites of the Taiwan Strait. The seasonal variation of PM_2.5_ concentration was quite consistent at all sampling sites. Higher PM_2.5_ concentrations were mostly observed in spring, winter, and fall (northeasterly winds) than in summer (southwesterly winds), indicating that atmospheric PM_2.5_ was mainly affected by meteorological condition. The highest PM_2.5_ at the both sides of the Taiwan Strait in winter and spring were likely due to the combination of the relatively elevated emissions from fossil fuel combustion and biomass burning as well as Asian dusts from northern China.

Results from the chemical analysis of PM_2.5_ indicated that the major water-soluble ionic species of PM_2.5_ were secondary inorganic aerosols (SIA), while the major metallic elements were crustal contents. Carbonaceous analysis results showed that primary organic carbons (POC) were always higher than secondary organic carbons (SOC) at the both sides of the Taiwan Strait. The ionic concentrations of PM_2.5_ at the west-side sites were approximately two times higher than those at the offshore and the east-side sites, which were adjacent to major emission sources, such as industrial boilers, heavy oil, and coal burning at the west-side sites. Results of the mass percentage of chemical composition of PM_2.5_ (Fig. 7) showed that the TP and PH sites had consistent concentration and chemical characteristics of PM_2.5_, suggesting that PM_2.5_ at the windward regions came mainly from clean marine air in all seasons. The chemical compositions of PM_2.5_ were consistent at the XM and KH sites since metro Xiamen and Kaohsiung have the largest industrial areas compared to other regions around the Taiwan Strait.

Under the transportation routes from the north, the concentrations of PM_2.5_ were always higher than those from the south. PM_2.5_ could be transported from North China, eastern coast of China, Korea Peninsula, or South Japan, associated to a higher contribution from coal burning in winter and fall. The transportation routes from the south could blow clean air masses from the ocean, which resulted in the dilution of PM_2.5_ in summer. We found that the major sources of PM_2.5_ at the west-side sites were anthropogenic sources. Similar source distributions were also observed at the east-side sites of the Taiwan Strait (Fig. 11). The results indicated that large human activities were generally existed at the coastal sites around the Taiwan Strait than those at the offshore islands. The major contributions of PM_2.5_ at the offshore islands were natural sources including oceanic spray, soil dusts, and biomass burning. The contributions of vehicular exhaust at the west-side sites were generally higher than those at the east-side sites, and followed by the offshore site around the Taiwan Strait. The contributions of secondary sulfate and nitrate increased from the west-side to the east-side sites around the Taiwan Strait, indicating an obvious transportation phenomenon of aged particles from the west-side to the east-side of the Taiwan Strait.

In order to further investigate the transportation of PM_2.5_, the sulfur and nitrogen oxidation ratios (SOR and NOR) were assessed to identify the aged particles around the Taiwan Strait (Fig. 9). The SOR and NOR value was higher at the west-side sites than those at the east-side and offshore sites, especially in fall, winter, and spring. High SOR and NOR in the areas at the west-side sites obtained in this study suggested that the formation of SO_4_^2−^ and NO_3_^−^ from SO_2_ and NO_x_ did occur in the atmosphere. Moreover, low NOR ratios at the TP and PH sites were mostly observed in spring and winter. The results indicated that low NO_x_ concentrations resulted in low conversion of NO_3_^−^ from NO_x_ at the windward regions around the Taiwan Strait.

## Methods

### Sampling Protocol

**Figure 12:**
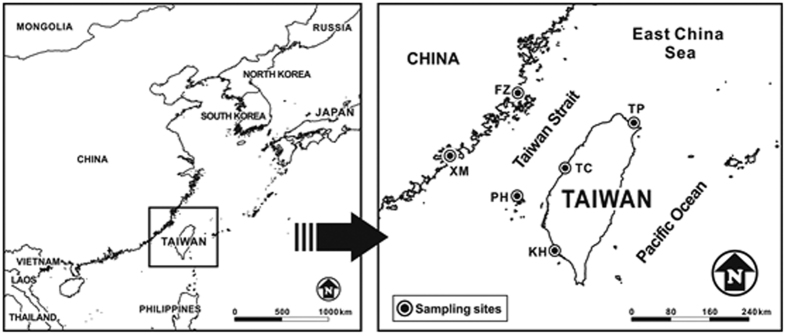
Location of PM_2.5_ sampling sites around the Taiwan Strait. The maps are created by a free computer program, IVA-GIS (http://www.diva-gis.org) and modified by CorelDRAW Graphics Suite X6 software (http://www.coreldraw.com/us/).

PM_2.5_ samples were collected at two sites (Xiamen and Fuzhou) in Fujian Province in southeastern China on the west-side of the Taiwan Strait, three sites (Kaohsiung, Taichung and Taipei) on the east-side of the Taiwan Strait, and one site at an offshore island (Penghu Islands) in the Taiwan Strait (see Fig. 12). The maps are created by a free computer program, IVA-GIS (http://www.diva-gis.org) and modified by CorelDRAW Graphics Suite X6 software. The sampling site in Xiamen City (XM), located at the main campus of Xiamen University (118°05′N, 24°26′E), was next to a street and likely to be influenced by the direct emissions of fine particles from vehicular exhausts. The sampling site in Fuzhou City (FZ) was situated at the campus of Fujian Normal University (119°18 N, 26°02′E), which is surrounded by neighboring residential buildings and traffics. The sampling site in New Taipei City (TP) was located at the Cape Santiago which was situated at Fulian Primary School at the northeastern coastline of Taiwan islands. The sampling site in Taichung City (TC) was located at the campus of Hungkuang University nearby a highway on the suburban of central Taiwan. The sampling site in Kaohsiung City (KH) was located at the campus of National Sun Yat-sen University on the suburban coastline of Kaohsiung City, the biggest industrial city in Southern Taiwan. The sampling of PM_2.5_ was simultaneously conducted using quartz fiber filters at six sampling sites from July 2013 to April 2015. PM_2.5_ was sampled by a high-volume sampler with a cascade impactor for continuous 24 hours from 9:00 am to 9:00 am of the sequential day at each site seven days per season^79^. In this study, high-volume samplers with same brand name were used to collect PM_2.5_ with the sampling flow rate of 1.4 m^3^/min. This sampling method was complied with the sampling method of NIEA A102.12A similar to USEPA Method IO-2.1. After sampling, the PM_2.5_ samples were analyzed for their chemical composition including water-soluble ions, metallic elements, and carbonaceous content. The *Chemical Analysis* and *Quality Assurance and Quality Control* are shown as Methods in Supplementary Information.

## Additional Information

**How to cite this article**: Li, T.-C. *et al.* Inter-comparison of Seasonal Variation, Chemical Characteristics, and Source Identification of Atmospheric Fine Particles on Both Sides of the Taiwan Strait. *Sci. Rep.* **6**, 22956; doi: 10.1038/srep22956 (2016).

## Supplementary Material

Supplementary Information

## Figures and Tables

**Table 1:** The concentrations of fine and coarse particles and their mass ratios at six sampling sites around the Taiwan Strait.

Sampling Sites	Sampling Periods	Sample Number	PM_2.5_ (μg/m^3^)	PM_2.5~10_ (μg/m^3^)	PM_10_ (μg/m^3^)	PM_2.5_/PM_10_ (%)
West-side (XM, FZ)	Summer	14	37.7	24.8	62.4	60.3
	Fall	14	42.1	35.1	77.2	54.5
	Winter	14	67.4	54.7	122.0	55.2
	Spring	14	78.6	51.0	129.6	60.6
Offshore island (PH)	Summer	7	15.1	17.8	32.9	46.0
	Fall	7	28.3	19.9	48.2	58.7
	Winter	7	28.2	23.3	51.5	54.8
	Spring	7	22.7	19.7	42.4	53.5
East-side (TP, TC, KH)	Summer	21	19.0	15.8	34.9	54.6
	Fall	21	35.5	30.1	65.6	54.2
	Winter	21	48.0	23.0	71.0	67.6
	Spring	21	33.8	28.5	62.3	54.3

**Table 2:** The neutralization factor (NF) of Ca^2+^, Mg^2+^, and NH_4_^+^ with SO_4_^2−^ and NO_3_^−^.

NF	West-side sites				Offshore site				East-side sites			
	Summer	Fall	Winter	Spring	Summer	Fall	Winter	Spring	Summer	Fall	Winter	Spring
NH_4_^+^	0.7	0.9	0.8	0.7	0.5	0.5	0.6	0.6	0.5	0.5	0.5	0.5
Ca^2+^	0.2	0.1	0.1	0.1	0.2	0.2	0.1	0.2	0.2	0.2	0.1	0.1
Mg^2+^	0.1	0.1	0.1	0.1	0.1	0.1	0.1	0.1	0.1	0.1	0.1	0.1

Unit: μeq m^−3^.

**Table 3:** The mass ratio of secondary inorganic aerosols (SIA) to water-soluble (WSI) and PM_2.5_ concentration during the sampling periods.

Seasons	Sampling Sites	SIA/WSI (%)	SIA/PM_2.5_(%)	Seasons	SIA/WSI (%)	SIA/PM_2.5_(%)
Summer	XM	63.6	23.0	Winter	78.7	39.0
	FZ	56.6	24.5		77.2	33.5
	PH	61.3	24.0		75.3	33.5
	TP	60.0	29.5		75.7	31.3
	TC	64.4	28.5		81.3	38.9
	KH	59.3	24.7		70.4	29.7
Fall	XM	75.2	35.2	Spring	82.6	34.2
	FZ	65.7	30.2		83.0	37.3
	PH	74.0	30.8		78.6	35.9
	TP	74.1	33.9		73.6	32.4
	TC	75.8	31.8		74.2	32.8
	KH	75.5	34.1		67.2	31.9

Secondary Inorganic Aerosols (SIA) = NO_3_^−^ + SO_4_^2−^+ NH_4_^+^; WSI: Water-soluble Ions.

**Table 4:** Comparison of the nss-NO_3_^−^, ss-NO_3_^−^, and ratio of nss-NO_3_^−^/NO_3_^−^ at the Taiwan Strait.

Sampling Sites	West-side Sites				Offshore Site				East-side Sites			
Species	Spring	Summer	Fall	Winter	Spring	Summer	Fall	Winter	Spring	Summer	Fall	Winter
nss-NO_3_^−^ (μg m^−3^)	6.1	3.2	3.0	5.8	2.6	0.9	2.3	2.9	2.4	1.2	1.7	4.2
ss-NO_3_^−^ (μg m^−3^)	1.7	1.1	1.2	2.1	0.6	0.4	0.7	0.6	1.4	0.7	0.9	1.5
nss-NO_3_^−^/NO_3_^−^ (%)	78.4	74.7	71.4	73.5	82.1	70.4	76.9	81.9	63.9	63.3	66.5	73.8

**Table 5:** Comparison of PM_2.5_ chemical composition during the sampling periods.

Sampling Sites	EC/TC	K/TC	NO3-/nss-SO_4_^2−^	TC/SO_4_^2−^	Reference
Xiamen	0.3	0.2	0.6	0.9	This study (2015)
Fuzhou	0.3	0.1	0.7	1.2	This study (2015)
Penghu	0.4	0.2	0.5	0.9	This study (2015)
Kaohsiung	0.4	0.1	0.6	0.8	This study (2015)
Taichung	0.4	0.2	0.8	0.9	This study (2015)
Taipei	0.4	0.2	0.7	0.8	This study (2015)
Fuzhou	0.2	0.02	0.4	1.0	Xu *et al.*^80^
Xiamen	0.2	0.1	0.5	1.2	Zhang *et al.*^81^
Xiamen	0.2	–	0.5	1.2	Zhao *et al.*^82^
Qingdao	0.2	0.1	1.0	1.5	Cao *et al.*^2^
Hong Kong	0.3	0.04	1.0	0.9	Cao *et al.*^2^
Shanghai	0.2	0.04	1.0	1.5	Cao *et al.*^2^
Seoul, Korea	0.3	0.03	0.9	1.7	Heo *et al.*^83^
Yokohama, Japan	0.3	0.02	1.1	1.5	Khan *et al.*^63^
Italy	0.1	–	1.9	2.4	Lonati *et al.*^84^

**Table 6:** The distribution of major chemical components of PM_2.5_ at the coastal sites around the Taiwan Strait and East China Sea.

Sampling Sites	Average Con.	SO_4_^2−^ (%)	NO_3_^−^ (%)	NH_4_^+^ (%)	Organic Materials (%)	Crustal Materials (%)	Elemental Carbon (%)	Reference
Xiamen	59.2	18.8	10.2	8.1	21.4	21.3	4.3	This study (2015)
Fuzhou	53.6	15.0	9.8	7.8	23.8	26.4	3.7	This study (2015)
Penghu	23.6	16.4	9.0	3.6	19.6	32.9	4.5	This study (2015)
Kaohsiung	41.8	18.1	8.9	4.6	19.5	24.6	5.5	This study (2015)
Taichung	35.6	16.7	9.9	4.1	19.3	24.4	5.7	This study (2015)
Taipei	24.9	17.7	10.4	4.0	22.1	28.2	5.3	This study (2015)
4 Coastal Sites of Fujian Province, China	55.1	19.8	9.7	10.6	30.2	4.7	2.9	Wu *et al.*^32^
Xiamen	74.2	17.0	10.0	9.0	34.0	18.0	7.0	Cao *et al.*^2^
Shanghai	139.4	15.0	12.0	10.0	32.0	21.0	6.0	Cao *et al.*^2^
Qingdao	134.8	16.0	14.0	11.0	31.0	18.0	4.0	Cao *et al.*^2^
Hong Kong	88.4	24.0	11.0	8.0	25.0	18.0	7.0	Cao *et al.*^2^
Seoul, Korea	37.6	15.3	13.8	9.8	30.2	7.4	7.7	Heo *et al.*^83^
Yokohama, Japan	37.6	18.4	4.7	11.0	21.8	NA	9.4	Khan *et al.*^63^

**Table 7:** The source apportionment of atmospheric PM_2.5_ during the sampling periods.

Sources	XM	FZ	PH	TP	TC	KH
Industrial Boilers	15.1 ± 0.8	8.5 ± 1.0	4.8 ± 1.3	7.6 ± 0.9	12.4 ± 0.9	14.8 ± 1.5
Petroleum Plants	6.7 ± 0.8	5.8 ± 1.5	4.0 ± 1.4	6.5 ± 0.3	10.5 ± 1.1	12.6 ± 1.0
Cement Plants	2.9 ± 0.8	3.8 ± 0.8	1.7 ± 1.8	5.8 ± 1.0	2.3 ± 0.7	4.2 ± 0.9
Secondary Nitrate	9.1 ± 1.6	9.8 ± 1.3	9.9 ± 1.1	9.1 ± 1.2	11.2 ± 1.5	11.5 ± 1.7
Secondary Sulfate	11.9 ± 1.6	11.6 ± 1.7	12.9 ± 1.2	13.1 ± 0.7	14.2 ± 0.7	14.5 ± 1.1
Gasoline Exhausts	7.5 ± 1.9	8.8 ± 2.4	3.4 ± 1.1	6.2 ± 2.5	7.6 ± 2.5	4.6 ± 1.2
Diesel Exhausts	6.9 ± 0.6	5.3 ± 1.3	8.0 ± 1.0	6.0 ± 0.6	4.2 ± 1.3	7.6 ± 0.5
Oceanic Spray	4.8 ± 1.8	7.8 ± 1.2	10.7 ± 1.1	7.0 ± 2.1	4.3 ± 1.3	5.0 ± 0.9
Biomass Burning	5.2 ± 0.5	6.7 ± 2.9	9.4 ± 1.5	5.5 ± 0.2	2.9 ± 0.3	2.9 ± 0.8
Soil Dusts	18.2 ± 1.6	17.8 ± 3.9	20.7 ± 2.3	16.1 ± 1.4	12.8 ± 0.4	13.1 ± 2.0
Steel Plants	4.2 ± 2.7	3.2 ± 2.2	1.2 ± 0.5	2.7 ± 0.6	2.9 ± 1.5	6.8 ± 2.5

Unit: Average (%) ± S.D.
