# Supplementary material for: Inter-comparison of Seasonal Variation, Chemical Characteristics, and Source Identification of Atmospheric Fine Particles on Both Sides of the Taiwan Strait
Source: Sci Rep. 2016 Mar 14;6:22956. doi: 10.1038/srep22956 (PMC4789734; doi:10.1038/srep22956)
Supplement: Supplementary Information [file srep22956-s1.pdf]

Inter-comparison of Seasonal Variation, Chemical Characteristics, and Source Identification of Atmospheric Fine Particles on Both Sides of the Taiwan Strait

Supplementary Information

Tsung-Chang Li[1], Chung-Shin Yuan[1*], Hu-Ching Huang[2], Chon-Lin Lee[2], Shui-Ping Wu[3], Chuan Tong[4]

[1]Institute of Environmental Engineering, National Sun Yat-sen University, Taiwan, Kaohsiung 804, R.O.C.
[2]Department of Marine Environment and Engineering, National Sun Yat-sen University, Taiwan, Kaohsiung 804, R.O.C.
[3]College of Environment and Ecology, Xiamen University, Xiamen 361005, P.R.C.
[4]School of Geographic Science, Fujian Normal University, Fuzhou, P.R.C.

* To whom all correspondence should be addressed

Tel: 886-7-5252000 Ext. 4409; Fax: 886-7-52524409;

E-mail: ycsngi@mail.nsysu.edu.tw

[*] Corresponding author. Tel: 886-7-5252000 ext 4409; Fax: 886-7-5254449;
E-*mail address*: ycsngi@mail.nsysu.edu.tw

Table S-1 Sampling location and environmental description for six sampling sites around the Taiwan Strait.

Sampling region	Sampling sites	Abbr.	Latitude	Longitude	Altitude (m)	Site description
East-side	Xiamen University	XM	24°26'08"	118°05'25"	21	suburban island
	Fujian Normal University	FZ	26°02'24"	119°18'85"	35	suburban resident
Offshore island	Penghu Islands	PH	23°33'41"	119°35'10"	25	offshore island
West-side	National Sun Yat-sen University	KH	22°37'36"	120°15'57"	20	suburban coastline
	Hungkuang University	TC	24°21'80"	120°58'22"	30	suburban roadside
	Cape Santiago in Taipei	TP	25°00'27"	122° 00'06"	10	remote coastline

XM: Xiamen; FZ: Fuzhou; PH: Penghu; KH: Kaohsiung; TC: Taichung; TP: Taipei

Transportation Routes of Air Masses

Figure S-1 illustrates the transportation routes of air masses toward the Taiwan Strait. The analysis of the back-trajectories was used to give insight into the potential contribution sources from different direction. We used the chemical characteristic trajectory statistical methods to investigate the transportation of $PM_{2.5}$ came from potential source regions by air masses. The corresponding trajectories could be clustered into eight major directions according to their source regions through which the air masses traveled to the target areas. The transportation routes of air masses during the sampling periods are described below, (1) NW: air masses originated from Mongolia; (2) NE: air masses transported from Japan Sea and Bohai Bay; (3) NEE: air masses mainly come from the West Pacific Ocean adjacent to East Japan; (4) SEE: air masses originated from the West Pacific Ocean adjacent to East Taiwan; (5) SE: air masses originated from the East Philippines; (6) S: air masses transported from the West Philippines; (7) SW: air masses transported from the South China Sea; (8) NWW: air masses come from the South China.

Table S-1 summarizes the eight trajectories of air masses blown from different directions and regions. The transportation routes of air masses in spring came mainly from NW (64%), and followed by SE (12%), SW (10%), NWW (10%), and NE (5%) at the east-side sites of the Taiwan Strait. At the west-side sites of the Taiwan Strait, the transportation routes of air masses came from NW and NE, which accounted for 71% and 29% of the total trajectories, respectively. At the offshore (PH) site of the Taiwan Strait, the transportation routes of air masses were originally blown from five directions, including NW (7%), NE (29%), NEE (7%), SEE (43%), and NWW (14%). In summer, the transportation routes of air masses mainly came from S (33%), and followed by SEE (31%), SE (17%), SW (14%), and NEE (5%) at the east-side sites of the Taiwan Strait. At the offshore (PH) site of the Taiwan Strait, the transportation routes of air masses came from SEE, S, and SW accounted for 43%, 29%, and 29% of

the total trajectories, respectively. At the west-side sites of the Taiwan Strait, the transportation routes of air masses were originally blown from SW (64%), SE (32%), and S (4%), respectively. In fall, the transportation routes of air masses mainly came from NE (52%), and followed by NEE (26%), NWW (10%), NW (7%), and SEE (5%) at the east-side sites of the Taiwan Strait. At the offshore (PH) site of the Taiwan Strait, the transportation routes of air masses mainly came from NE, NEE, and NW, accounted for 56%, 33%, and 11%, respectively, of the total trajectories. At the west-side sites of the Taiwan Strait, the transportation routes of air masses were originally blown from NE (68%), NW (21%), and NE (11%), respectively. In winter, the transportation routes of air masses mainly came from NEE (33%), NE (24%), SEE (12%), SW (10%), NWW (10%), SE (7%), and S (5%) at the east-side sites of the Taiwan Strait. At the offshore (PH) site of the Taiwan Strait, there was only one transportation route of air masses from NW. At the west-side sites of the Taiwan Strait, the transportation routes of air masses were originally blown from NE (61%), NEE (25%), SEE (11%), and NW (4%), respectively.

The level of atmospheric $PM_{2.5}$ concentration was highly affected by meteorological condition, thus $PM_{2.5}$ concentrations in spring and winter was much higher than those in fall and summer. Moreover, backward trajectories showed that the concentrations of $PM_{2.5}$ blown from the north were generally higher than those blown from the south. Backward trajectory simulation results indicated that $PM_{2.5}$ could be transported from North China, eastern coast of China, Korea Peninsula, or South Japan.

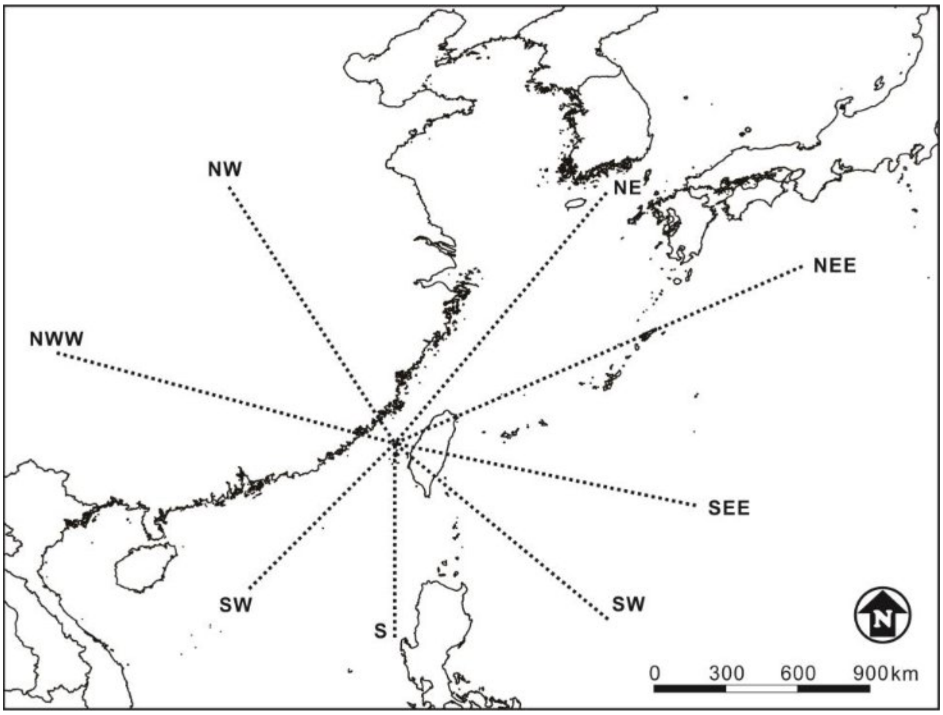

Figure S-1 Transportation routes of air masses toward the Taiwan Strait during the sampling periods. (NW: Mongolia; NE : Japan Sea and Bohai Bay; NEE : Pacific Ocean near Japan; SEE : Pacific Ocean near Taiwan; SE : East Philippines; S : West Philippines; SW : South China Sea; NWW : South China). The maps are created by a free computer program,D IVA-GIS (http://www.diva-gis.org) and modified by CorelDRAW Graphics Suite X6 software, (http://www.coreldraw.com/us/).

Table S-2 The frequency of air masses transported toward the Taiwan Strait during the sampling periods.

**Direction**	**Seasons**	**NW**	**NE**	**NEE**	**SEE**	**SE**	**S**	**SW**	**NWW**
East-side	Spring	64%	5%			12%		10%	10%
	Summer			5%	31%	17%	33%	14%	
	Fall	7%	52%	26%	5%				10%
	Winter		24%	33%	12%	7%	5%	10%	10%
Offshore island	Spring	7%	29%	7%	43%				14%
	Summer				43%		29%	29%	
	Fall	11%	56%	33%					
	Winter	100%							
West-side	Spring	71%	29%						
	Summer					32%	4%	64%	
	Fall	21%	68%	11%					
	Winter	4%	61%	25%	11%				

Spatial variations of $PM_{2.5}$ for Various Transportation Routes

The backward trajectory of air parcel is a widely used simulation scheme that plots the trajectory of a single air parcel from a given location and height above ground over a period of time. In this study, we simulated the potential contribution sources from different spatial trajectories to investigate the transport of $PM_{2.5}$ toward the Taiwan Strait. The results of backward trajectory analysis were not shown in this manuscript. Preliminary results showed that the highest $PM_{2.5}$ concentrations were mostly observed on the trajectories from NW directions at both sides of the Taiwan Strait in spring, fall, and winter, particularly at the west-side sites of the Taiwan Strait in spring (see Table S-3).

Tao et al[1]. reported that the highest $PM_{2.5}$ concentrations in winter and spring are most likely due to a combination of the relatively elevated emissions from fossil fuel (e.g. coal) combustion and biomass burning (e.g. woods) for space heating as well as stagnant meteorological conditions, such as low mixing layer, which limits the dispersion of air pollutants in northern China. In summer, the highest $PM_{2.5}$ concentrations were generally observed on the trajectories from SW directions at the west-side sites, and trajectories from SEE directions at the east-side sites and the offshore island site. The results indicated that the major sources of $PM_{2.5}$ were probably from southern China and northern Indochina Peninsula. The air masses could transport $PM_{2.5}$ across the Taiwan Strait toward the east-side sites. Additionally, this study found several high concentrations of $PM_{2.5}$ came from Pacific Ocean adjacent to the Taiwan Strait.

Different backward trajectories could blow air pollutants from various upwind regions (i.e. northern China, Korea Peninsula, Japan Islands, Indochina Peninsula and etc...), which also have special chemical characteristics in different seasons. Moreover, the prevailing winds were blown northeasterly during the period of Northeastern

Monsoons in the Taiwan Strait in winter and spring. Northeastern Monsoon might blow air pollutants northerly from mainland China, Korea Peninsula, and Japan Islands toward both sides of the Taiwan Strait. However, the southwestern Monsoons might transport air pollutants from the Indochina Peninsula in late spring and summer

Table S-3 The concentrations of $PM_{2.5}$ for various transportation routes.

Seasons	Sampling Sites	**NW**	**NE**	**NEE**	**SEE**	**SE**	**S**	**SW**	**NWW**
Spring	East-side	**36.19**	30.91	-	-	27.53	-	27.16	33.95
	Offshore	**23.91**	22.63	19.37	23.05	-	-	-	22.73
	West-side	**81.31**	71.76	-	-	-	-	-	-
Summer	East-side	-	-	17.73	**19.53**	18.71	19.46	17.59	-
	Offshore	-	-	-	**16.46**	-	14.02	14.22	-
	West-side	-	-	-	-	33.88	36.47	**39.61**	-
Fall	East-side	**37.65**	35.13	35.64	33.61	-	-	-	36.66
	Offshore	**30.13**	28.43	27.44	-	-	-	-	-
	West-side	**44.22**	41.60	40.73	-	-	-	-	-
Winter	East-side	-	47.93	47.61	**48.05**	46.15	46.44	49.33	50.34
	Offshore	**28.19**	-	-	-	-	-	-	-
	West-side	**55.03**	54.32	53.04	53.30	-	-	-	-
Annual	East-side	**37.99**	36.92	33.66	33.73	30.80	32.95	31.36	40.32
	Offshore	**27.41**	25.53	23.41	19.76	-	14.02	14.22	22.73
	West-side	**59.95**	56.13	46.89	53.30	33.88	36.47	39.61	-

Unit: $\mu g\ m^{-3}$

Method

Chemical Analysis

After sampling $PM_{2.5}$, the quartz fiber filters were temporarily stored at 4℃ to conserve the chemical stability, and were then sent back to the Air Pollution Laboratory of the Institute of Environmental Engineering at National Sun Yat-sen University for further chemical analysis. All $PM_{2.5}$ filters were divided into four identical parts prior to the chemical analysis. One quarter of the quartz fiber filter was analyzed for water-soluble ionic species of $PM_{2.5}$ by means of ion chromatography (DIONEX, Model DX-120). The concentrations of the major anions (F^-, Cl^-, SO_4^{2-}, and NO_3^-) and cations (NH_4^+, K^+, Na^+, Ca^{2+}, and Mg^{2+}) were measured. The filters analyzed for ionic species were put into 15-ml bottles for each sample. Distilled de-ionized water was added into each bottle for ultrasonic vibration of approximately 60 minutes.

Another quarter of the quartz fiber filter was digested by microwave digestion method in a 30 mL mixed acidic solution (HNO_3: HCl = 3:7) by heating it up to 150–200°C for 2 hours, and then diluted to 25 ml with distilled de-ionized water (D.I. H_2O) for metallic elements analysis. During the digestion process, D.I. H_2O was added to the residual solution for two or more times in order to eliminate the acidic content of the digestion solution. Thirteen metallic species of $PM_{2.5}$ including Na, Ca, Al, Fe, Mg, K, Zn, Cr, Ti, Mn, Ni, and Pb were analyzed with an inductively coupled plasma-atomic emission spectrometer (ICP-AES) (Perkin Elmer, Model Optima 2000DV).

The carbonaceous contents (elemental, organic, and total carbons) of $PM_{2.5}$ were measured with an elemental analyzer (EA) (Carlo Erba, Model 1108). Before sampling, the quartz fiber filters had to be pre-heated at 900°C for 1.5 h to expel the potential impurities. This preheating procedure minimized the background carbon in the quartz fiber filters and matrix, which would interfere with the analytical results,

leading to an overestimation of the carbonaceous contents of $PM_{2.5}$. The elemental analyzer was operated using the procedure of oxidation at 1020°C and that of reduction at 500°C, with continuous heating for 15 min. Additionally, one eighth of the quartz fiber filters was heated in advance by hot nitrogen gas (340-345°C) for 30 min to expel the organic carbon (OC) fraction, after which the amount of elemental carbon (EC) was determined. Another eighth of the quartz fiber filters was analyzed without heating, and the carbon thus characterized as total carbon (TC). The amount of organic carbon was then estimated by subtracting the elemental carbon from total carbon. Although the most widely used method, thermal analysis, was used to determine the carbon contents in ambient aerosols, a charring formation error from sample preheating was not taken into account for correction, and this artifact might cause in the overestimation of EC and the underestimation of OC[2].

In this study, quartz fiber filters were carefully handled and placed on the PM_{10} samplers to prevent potential cracking during the sampling procedure. After sampling, aluminum foil was used to cover the quartz filters which were temporarily stored at 4 °C and transported back to the laboratory for chemical analysis. Background contamination was routinely monitored by using operational blanks (unexposed filters) which were processed simultaneously with field samples. In this study, the background contamination was insignificant and can thus be ignored. At least 10% of the samples were analyzed by spiking with a known amount of metallic and ionic species to determine the recovery efficiencies. The results of these recovery efficiency tests indicated the range of recovery efficiency among every 10 samples varied between 96 and 103%. In addition, the reproducibility test varied between 97 and 104% for all the chemical species.

Quality Assurance and Quality Control

Quality assurance and quality control (QA/QC) for both $PM_{2.5}$ sampling and the chemical analysis were conducted in this study. Prior to sampling, the flow rate of each $PM_{2.5}$ sampler was carefully calibrated with a film flow meter (SENSIDYNE MCH-01). The quartz fiber filters were handled with care, so as to prevent potential cracking during the sampling procedure, as they were placed on the $PM_{2.5}$ samplers. After sampling, aluminum foil was used to fold the quartz fiber filters, which were then temporarily stored at $4^{o}C$ and transported back to the central laboratory for chemical analysis. The sampling and analytical procedures were similar to those described in previous studies[3-7]. Both field and transportation blanks were undertaken for $PM_{2.5}$ sampling, while reagent and filter blanks were applied for chemical analysis. The determination coefficient (R^2) of the calibration curve for each chemical species was required to be higher than 0.995. Background contamination was routinely monitored by using operational blanks (unexposed filters); these were processed simultaneously with the field samples. The background interference was insignificant and could thus be ignored in this study. At least 10% of the samples were analyzed by spiking with a known amount of metallic and ionic species to determine their recovery efficiencies.

Chemical Transformation of SO_2 and NO_X

Sulfate and nitrate are the major components contained in atmospheric fine particles. To determine the degree of atmospheric transformation of SO_2 to SO_4^{2-} and NO_x to NO_3^-, the sulfur and nitrogen oxidation ratios (i.e. SOR and NOR) were employed, which are defined as follows [8-10]:

$$SOR = \frac{S_{nss-SO_4^{2-}}}{S_{nss-SO_4^{2-}} + S_{SO_2}} \quad (1)$$

$$NOR = \frac{N_{NO_3^-}}{N_{NO_3^-} + N_{NO_2}} \quad (2)$$

where nss-SO_4^{2-} is the excess non-sea salt sulfate, calculated by subtracting the amount of SO_4^{2-} in seawater from that of SO_4^{2-} in atmospheric fine particles[10-11] ; the units of nss-SO_4^{2-}, S_{SO_2}, $N_{NO_3^-}$, and N_{NO_2} are neq/m^3. The average concentrations of SO_2 and NO_x during each sampling period were obtained from the ambient air quality monitoring stations situated near the $PM_{2.5}$ sampling sites in the areas around the Taiwan Strait.

Chemical Mass Balanced (CMB) Receptor Model

The source apportionment of ambient $PM_{2.5}$ was assessed using a receptor model based on the principle of chemical mass balance (CMB). Since the detailed descriptions of CMB receptor model (e.g., CMB 8.0) are available elsewhere, only a brief summary is presented below. The CMB receptor model uses the emission profiles of prominent sources to estimate their contribution to a specific receptor. It is assumed that the total concentration of a particular chemical species at the receptor is the linear summation of each individual contribution from various sources. The CMB receptor model uses the results of the least-square regression analysis of the aerosol chemical composition to resolve the most appropriate contributions of source apportionment. Therefore, this model consists of a least-square solution to a set of linear equations. This solution expresses each receptor concentration of a chemical species as a linear summation of the products of source profiles and source contributions. Source profiles (the fractional amount of each species in the emissions from each source type) and receptor concentrations, each with realistic uncertainty estimates, serve as the input data to the CMB receptor model. The model output consists of the contribution from each source type to the total ambient aerosol particle mass, as well as to individual chemical species concentration. The CMB8 model are evaluated by using several fit indices, such as R^2 ($\geq$ 0.8), χ^2 ($\leq$ 4.0), T statistics ($\geq$ 2.0), and the percentage of mass accounted for 80-120%. The source profiles used in this study were reported by USEPA, Southern California Air Quality Study, and the researches

studied the chemical composition of $PM_{2.5}$ from local prominent sources in Taiwan. Table S-3 lists the source profiles which were used for apportioning the sources of $PM_{2.5}$ sampled around the Taiwan Strait.

Table S-3 Source Profiles of $PM_{2.5}$ used for chemical mass balance receptor modeling.

	Code	Source Profile	Researchers
SCT004	PBPRI1	Petroleum cracking Plant	U.S. EPA. 1991
SCT007	PP004	Industrial Boilers (Oil)	Cheng *et al.*, 2000
SCT008	PP005	Industrial Boilers (Coal)	Cheng *et al.*, 2000
SCT009	PETRO1	Petroleum Industry	U.S. EPA. 1991
SCT010	STEEL1	Steel Industry	Chiang *et al.*, 1993
SCT011	STEEL2	Coke Plant	Chiang *et al.*, 1993
SCT012	STEEL3	Sinter Plant	Chiang *et al.*, 1993
SCT013	STEEL4	Electric Arc Furnace	Yuan *et al.*, 2003
SCT020	CEMENT	Cement Industry	Chiang *et al.*, 1993
SCT023	VEHICLE2	Vehicular Exhausts	J.C Chow. 1991
SCT024	VEHICLE3	Diesel Exhausts	J.C Chow. 1991
SCT025	DUST1	Paved Road dust in South Taiwan	Cheng *et al.*, 1998
SCT026	DUST2	Paved Road dust in Central Taiwan	Cheng *et al.*, 1998
SCT027	DUST3	Paved Road dust in South Taiwan	Yuan *et al.*, 1991
SCT028	DUST4	Paved Road dust in Central Taiwan	Chiang *et al.*, 1993
SCT029	DUST5	Unpaved Road dust in Central Taiwan	Chiang *et al.*, 1993
SCT031	SOIL1	Soil Dusts	U.S. EPA. 1991
SCT033	MARIN1	Marin in Central Taiwan	Cheng *et al.*, 1998
SCT034	MARIN2	Marin in South Taiwan	Chen *et al.*, 1998
SCT035	VB001	Biomass Burning	Cheng *et al.*, 1999
SCT037	SO4	Secondary Sulfate	Wang *et al.*, 2006
SCT038	NO2	Secondary Nitrate	Wang *et al.*, 2006
SCT039	STONE	Stone Processing Industry	Li *et al.*, 2013
SCT040	CEMENT2	Cement Industries	Li *et al.*, 2013
SCT041	CERM1	Ceramic Plants	Li *et al.*, 2013
SCT042	CERM2	Tile Industries	Li *et al.*, 2013
SCT043	COAL	Coal Burning	Li *et al.*, 2013
SCT044	COAA	Coal Ash	Li *et al.*, 2013
SCT045	SOIL2	Fugitive Dusts	Li *et al.*, 2013
SCT046	VB002	Biomass Burning	Li *et al.*, 2013
SCT047	CONST	Construction Dusts	Li *et al.*, 2013
SCT048	DUST6	Road Dusts	Li *et al.*, 2013

The source profiles used in this study were mainly obtained from the researcher's findings of the chemical composition of $PM_{2.5}$ emitted from various emission sources. Only limited source profiles are referred from USEPA and Southern California Air Quality Study, and local emission source profiles.

Background of Coastal and Offshore Regions around the Taiwan Strait

Fuzhou Region

Fuzhou City (25°15′–26°39′N and 118°08′–120°31′E), the political and economic center of Fujian province in southeast China, is one of the fastest industrializing and urbanizing regions in the western coastal region of the Taiwan Strait, with an area of 11,968 km^2 and a population of approximately 7 million. The annual average relative humidity (RH), precipitation, and wind speed in Fuzhou are 77%, 1342.5 mm, and 2.8 m/s, respectively. The highest and the lowest monthly ambient temperature are 28.6 °C (July) and 10.5 °C (January), respectively, with the annual average of 19.6 °C. The prevailing wind directions are southerly in summer and northeasterly in the rest seasons. Fuzhou City is situated in the center of a basin surrounding by steep mountains. The atmosphere in Fuzhou is relatively stable with high frequency of calm winds and temperature inversion in spring and winter. Due to its geographic location and climate features, atmospheric particulate matters are difficult to disperse and easy to accumulate gradually in the cold seasons. Fuzhou is increasingly facing its air pollution problems with rapid economic development. For instance, the hazy days occurred now days are much more often than those in the past decade. The rising number of traffic vehicles in Fuzhou has been regarded as one of main reasons for the deterioration of ambient air quality in urban area.

Xiamen Region

Xiamen Island is located at the southeastern coast of China where its southwest is the Jiulong River estuary and its southeast facing Kinmen Islands is adjacent to the Taiwan Strait. Xiamen Harbour on the northwestern side of the island is the biggest cargo terminal in Fujian Provence. With rapid development of industries, ports, offshore transport and mariculture, many toxic organic compounds are discharged to

the ocean, seriously affecting the marine ecological environment and causing great health risks to the living beings in the region.

Penghu Islands

The Penghu Islands is an offshore islands located at the southeastern coast of the Taiwan Island. The Penghu Islands have a total area of 128.0 km^2, which has the subtropical weather being mainly influenced by East Asian Monsoons. The potential air pollution sources at the Penghu Islands include an oil-fired power plant, a few construction sites, and limited motor vehicles. In winter and spring, the prevailing winds are blown northeasterly during the period of the Northeastern Monsoons. Long-range transportation is superior to local sources at the Penghu Islands. We have added the background of potential sources in the Penghu Islands in the supplementary file per request. The major contributions of $PM_{2.5}$ were soil dusts (20.65%), and followed by secondary sulfate (12.93%), oceanic spray (10.69%), and secondary nitrate (9.88%) at the PH site. The contributions of anthropogenic source (i.e. industrial source, petroleum plants, cement plants, and steel plants) at the offshore site were lower than those at the west-side sites and the east-side sites. The Lowest contribution of anthropogenic source (i.e. industrial source, petroleum plants, and secondary aerosol) and the highest contribution of natural source (i.e. soil dusts, oceanic spray, and biomass burning were observed at the offshore island site rather than those at both side sites of the Taiwan Strait. The results of CMB receptor modeling simulations were consistent with potential sources of $PM_{2.5}$ at the offshore islands.

Kaohsiung Region

The sampling site in Kaohsiung City (KH) was located at the biggest industrial city in Southern Taiwan. Kaohsiung City with its high percentage (6-8%) of poor air quality (PSI>100; Pollutants Standard Index >100), has been officially announced by the

Taiwan EPA as the worst air quality region among seven AQZs (Air Quality Zones) in Taiwan. Currently, there are more than 1.5 million automobiles and motorcycles and a total of 1,911 factories, including three utility power plants, two cement mills, an integrated steel plant, nine sizable iron works and 14 petrochemical plants in metro Kaohsiung (153 km^2). Moreover, Kaohsiung City is surrounded by four heavily polluted industrial parks. Among the particulate matter, fine particulates ($PM_{2.5}$) are one of the major particulates in the atmosphere.

Taichung Region

Taichung City located in the Taichung Basin is surrounded by Central Ranges to the east and Tadu Hill to the west. The vehicular emission is the most important source of atmospheric particles at an urban site, and followed by crustal materials, biomass burnings, industrial emissions, and marine spray. The prevailing wind comes from the north in fall and spring and from the southwest in summer. Under the northerly flow conditions, high PM_{10} days (HPD; $PM_{10} > 150$ µg m^{-3}) usually occur in the urban city. The calm condition and local circulation such as land-sea breeze, accompanying with Northeastern monsoon in HPD made the air sweep the pollutant source along the coast to the urban site, resulting in high PM_{10} concentrations.

Taipei Region

Cape Santiago is located at the northeastern tip of the Taiwan Island, which allows it as a background sampling site under northeasterly monsoons without the interferences of local sources. There were very few researches conducted at the northern tip of Taiwan coastal areas.

REFERENCES

1. Tao, J. et al. Chemical composition of $PM_{2.5}$ at an urban site of Chengdu in southwestern China. *ADV ATMOS SCI 30*, 1070-1084 (2013).
2. Lin, J.J. Characterization of the major chemical species in $PM_{2.5}$ in the Kaohsiung City, Taiwan. *Atmos Environ 36,* 1911-1920 (2002).
3. Yuan, C.S. et al. Correlation of Atmospheric Visibility with Chemical Composition of Kaohsiung Aerosols. *Atmos Res 82*, 663-679 (2006).
4. Cheng M.T. & Tsai, Y.I. Characterization of visibility and atmospheric aerosols in urban, suburban, and remote areas. *Sci Total Environ 263,* 101-114 (2000).
5. Lin, J.J. Characterization of the major chemical species in $PM_{2.5}$ in the Kaohsiung city, Taiwan. *Atmos Environ 36*, 1911-1920 (2002).
6. Tsai, H.H., Ti, T.H., Yuan, C.S., Hung, C.H. & Lin, C. Effects of sea-land breezes on the spatial and temporal distribution of gaseous air pollutants around the coastal region of southern Taiwan. *J. Environ. Eng. Manage 18*, 387-396 (2008).
7. Witz, S. et al. Rapid loss of particulate nitrate, chloride and ammonium on quartz fiber filters during storage. *J Air Waste Manag Assoc 40*, 53-61 (1990).
8. Colbeck, I. & Harrison R.M. Ozone-secondary aerosol visibility relationships in northwest England. *Sci Total Environ 34,* 87-100 (1984).
9. Ohta, S. & Okita, T. A chemical characterization of atmospheric aerosol in Sapporo. *Atmos Environ 24*, 815-822 (1990).
10. Chow, J.C. et al. Descriptive analysis of $PM_{2.5}$ and PM_{10} at regionally representative locations during SJVAQS/AUSPEX. *Atmos Environ 30*, 2079-2112 (1996).
11. Xing, L. et al. Seasonal and spatial variability of the OM/OC mass ratios and high regional correlation between oxalic acid and zinc in Chinese urban organic aerosols. *Atmos Chem Phys 13*, 4307–4318 (2013).
12. Wang, X.H., Bi, X.H., Sheng, G.Y. & Fu, J.M. Chemical Composition and Sources

of PM_{10} and $PM_{2.5}$ Aerosols in Guangzhou, China. *Environ Monit Assess 119*, 425–439 (2006).

13. Li, T.C., Chen, W.H., Yuan, C.S., Wu, S.P. & Wang, X.H. Diurnal variation and chemical characteristics of atmospheric aerosol particles and their source fingerprints at Xiamen Bay. *AEROSOL AIR QUAL RES 13*, 596-607 (2013).
